# Cancerous Inhibitor of PP2A Silencing Inhibits Proliferation and Promotes Apoptosis in Human Multiple Myeloma Cells

**DOI:** 10.1155/2016/6864135

**Published:** 2016-04-06

**Authors:** Xi Yang, Yaping Zhang, Hong Liu, Zenghua Lin

**Affiliations:** Department of Hematology, Affiliated Hospital of Nantong University, Nantong 226001, China

## Abstract

Multiple myeloma is the second most prevalent type of blood cancer, representing approximately 1% of all cancers and 2% of all cancer deaths. There is therefore a strong need to identify critical targets in multiple myeloma neoplasia and progression. Cancerous inhibitor of PP2A (CIP2A) is a human oncoprotein that regulates cancer cell viability and anchorage-independent growth and induces apoptosis. The present study investigated CIP2A function in the human multiple myeloma cell lines RPMI-8226 and NCI-H929 to determine whether it can serve as a potential therapeutic target.* CIP2A* was silenced in the cells by transfection of short interfering RNA and cell proliferation and apoptosis were evaluated by a tetrazolium salt-based assay and flow cytometry, respectively.* CIP2A* knockdown inhibited proliferation and induced apoptosis in RPMI-8226 and NCI-H929 cells and decreased the phosphorylation of phosphoinositide 3-kinase (PI3K) p85, AKT1, and mammalian target of rapamycin (mTOR) without affecting total protein levels. Treatment of* CIP2A*-depletion cells with insulin-like growth factor 1 decreased the effects of* CIP2A* inhibition on cell viability and apoptosis. These results indicate that CIP2A modulates myeloma cell proliferation and apoptosis via PI3K/AKT/mTOR signaling and suggest that it can potentially serve as a drug target for the treatment of multiple myeloma.

## 1. Introduction

Multiple myeloma—also known as plasma cell myeloma, myelomatosis, or Kahler's disease—is a cancer of plasma cells, a type of white blood cell that produces antibodies [1]. According to the National Cancer Institute Surveillance, Epidemiology, and End Results Program (http://seer.cancer.gov/), the number of new multiple myeloma cases is 6.3 per 100,000 men and women per year; mortality rate is 3.3 deaths per 100,000 men and women per year; and the 5-year survival rate is 46.6%. Multiple myeloma is the second most prevalent blood cancer (10%) after non-Hodgkin's lymphoma [2] and represents approximately 1% of all cancers and 2% of all deaths from cancer. It is therefore necessary to identify critical targets in multiple myeloma neoplasia and progression so that effective treatments can be developed.

The phosphoinositide 3-kinase (PI3K) signaling pathway mediates multiple myeloma cell proliferation and apoptosis and its activation increases with progression of the disease [3]. AKT is a downstream effector of PI3K that can be deactivated by the protein phosphatase 2A (PP2A) complex via dephosphorylation of Thr308 and Ser473 [4]. Activated AKT modulates the phosphorylation status of various substrates involved in cell survival, cell cycle progression, and cellular growth, including mammalian target of rapamycin (mTOR) [5]. PI3K/AKT/mTOR signaling regulates a broad range of cellular processes including survival, proliferation, growth, metabolism, angiogenesis, and metastasis and is hyperactivated or dysregulated in many cancer types [6]. The regulation of the PI3K/AKT/mTOR pathway is not fully understood with respect to multiple myeloma; however, in most cell types, it is controlled by phosphatases such as PP2A [7, 8]. For example, PP2A inhibition activates PI3K/AKT signaling through regulating the phosphorylation of AKT at Ser473 in SV40ST-induced human cancer cell transformation [8].

Cancerous inhibitor of PP2A (CIP2A)—also known as KIAA1524 or p90 tumor-associated antigen—is a human oncoprotein that is overexpressed in human neck and head carcinomas as well as breast, colon, and gastric cancers [5, 9–12]. Inhibiting CIP2A decreases cancer cell viability and anchorage-independent growth and induces apoptosis [5, 9]. In human malignancies, CIP2A stabilizes c-myc by inhibiting PP2A-mediated myc dephosphorylation at Ser62 [9]. In addition to blocking c-myc degradation, CIP2A expression is regulated by a positive feedback loop involving c-myc [10].

The precise function of CIP2A in human multiple myeloma has never been reported. The present study investigated the role of CIP2A in cell proliferation and apoptosis in the human multiple myeloma cell lines RPMI-8226 and NCI-H929 and its potential regulation of PI3K/AKT/mTOR signaling. The results demonstrate that CIP2A inhibition suppresses proliferation and induces apoptosis in RPMI-8226 and NCI-H929 cells while inactivating the PI3K/AKT/mTOR pathway. These findings provide novel insight into the roles of CIP2A in multiple myeloma progression and suggest that CIP2A can serve as a target for therapeutic agents in the treatment of this disease.

## 2. Materials and Methods

### 2.1. Cell Culture

The human multiple myeloma cell lines RPMI-8226 and NCI-H929 were purchased from the American Type Culture Collection (Manassas, VA, USA) and grown in RPMI-1640 medium supplemented with 10% (v/v) fetal bovine serum (FBS). The culture was maintained at 37°C in a 5% CO_2_ atmosphere.

### 2.2. Transfection and Insulin-Like Growth Factor 1 (IGF-1) Treatment

The short interfering RNA (siRNA) used to inhibit* CIP2A* expression was obtained from Jima Biotech (Suzhou, China). The following double-stranded oligonucleotides were used: CIP2A, 5′-CUG UGG UUG UGU UUG CAC UTT-3′, and scrambled, 5′-UUC UCC GAA CGU GUC ACG UTT-3′. For transfection, 2*∗*10^5^ cells were seeded in culture plates and transfected with* CIP2A* siRNA (si-CIP2A) or scrambled siRNA (si-Scr) using Lipofectamine 2000 (Invitrogen, Carlsbad, CA, USA) according to the manufacturer's instructions. After 48 h, cells were collected for analysis. IGF-1 (Sigma-Aldrich, Darmstadt, Germany) was used at a concentration of 100 ng/mL.

### 2.3. RNA Extraction and Quantitative Real-Time PCR (qRT-PCR)

Total RNA was extracted from cell samples using TRIzol reagent according to the manufacturer's protocol. RNA was reverse transcribed into cDNA using PrimeScript RT reagent kit with gDNA Eraser (Takara Bio, Dalian, China) in a 20 *μ*L reaction according to the manufacturer's protocol. Equal amounts of cDNA were used as template for qRT-PCR to detect the level of* CIP2A* expression relative to that of actin (endogenous control), which was quantitated on an Mx3000P Real-Time PCR System using the SYBR Premix Ex Taq II PCR kit (Takara Bio) and the following primers: actin-F, 5′-ACT TCA CAT CAC AGC TCC CC-3′, and actin-R, 5′-GAA TAT AAT CCC AAG CGG TTT G-3′, and CIP2A-F, 5′-CTG GTG AGA TAA TCA GCA ATT T-3′, and CIP2A-R, 5′-CGA AAC ATT CAT CAG ACT TTT CA-3′. Experiments were performed in duplicate and repeated twice. Fold induction of gene expression was calculated using the 2^−ΔΔCt^ method.

### 2.4. Cell Viability Assay

Cell viability was assessed with the [3-(4,5-dimethylthiazol-2-yl)-5-(3-carboxymethoxyphenyl)-2-(4-sulfophenyl)-2H-tetrazolium (MTS) assay 24 h after transfection of si-CIP2A of si-Scr. Cells were seeded in 96-well plates in RPMI-1640 medium supplemented with 10% FBS at a density of 2 × 10^3^ cells/well and cultured for 48, 72, and 96 h. Viability was measured using the CellTiter 96 AQueous One Solution Cell Proliferation Assay kit (Promega, Madison, WI, USA) according to the manufacturer's instructions. Briefly, 20 *μ*L CellTiter 96 AQueous One Solution reagent was added to each well, followed by incubation for 3 h at 37°C. The absorbance at 490 nm was measured using a Model 680 microplate reader (Bio-Rad, Hercules, CA, USA). Each experiment was carried out with six replicates and was repeated three times.

### 2.5. Apoptosis Assay

Apoptosis was determined using an apoptosis detection kit (Multisciences, Hangzhou, China) according to the manufacturer's instructions. Briefly, cells were digested with trypsin and collected by centrifugation at 2000 rpm for 5 min. Pellets were washed twice with phosphate-buffered saline (PBS) and centrifuged at 2000 rpm for 5 min; 1–5 × 10^5^ cells were collected and resuspended in 500 *μ*L binding buffer. A 5 *μ*L volume of annexin V-fluorescein isothiocyanate and 5 *μ*L propidium iodide were added to the suspension at room temperature and away from light for 15 min. Cells were sorted within 1 h by flow cytometry using an Accuri C6 instrument (BD Biosciences, Franklin Lakes, NJ, USA).

### 2.6. Western Blot Analysis

Total protein lysates were extracted from cells and equal amounts of total protein were resolved by 10% sodium dodecyl sulfate polyacrylamide gel electrophoresis and transferred to a nitrocellulose membrane. Nonspecific binding was blocked with 5% milk in tris-buffered saline with Tween 20, and membranes were incubated overnight at 4°C with primary antibodies against the following proteins: CIP2A (1 : 1000), AKT1 (1 : 2000), phosphorylated AKT1 (p-AKT1) (Ser473) (1 : 1500), PI3K p85 (1 : 500), p-PI3K p85 (Tyr458) (1 : 1000), mTOR (1 : 1000), p-mTOR (Ser2448) (1 : 2000), and poly(ADP-ribose) polymerase (PARP, 1 : 1500) (all from Cell Signaling Technology, Danvers, MA, USA). Membranes were then incubated with horseradish peroxidase-conjugated secondary antibody and protein bands were detected by enhanced chemiluminescence (Thermo Scientific, Waltham, MA, USA).

### 2.7. Statistical Analysis

Data are expressed as the mean ± SD and were analyzed using SPSS v. 19.0 software (SPSS Inc., Chicago, IL, USA). Differences between groups were assessed using Student's *t*-test. *P* < 0.05 was considered statistically significant.

## 3. Results

### 3.1. siRNA Knockdown of CIP2A Expression

**Figure 1:**
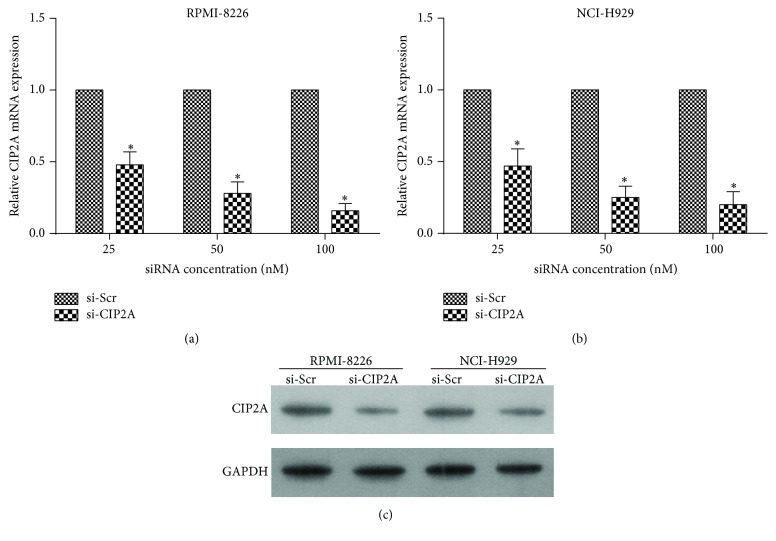
CIP2A expression following* CIP2A* knockdown. ((a) and (b))* CIP2A* mRNA expression level in RPMI-8226 (a) and NCI-H929 (b) cells after transfection of indicated concentrations of si-CIP2A or si-Scr, as detected by qRT-PCR. Data are expressed as mean ± SD. ^*∗*^ *P* < 0.05. (c) CIP2A protein expression after 100 nM si-CIP2A or si-Sci transfection, as determined by western blotting. Glyceraldehyde-3-phosphate dehydrogenase (GAPDH) served as a loading control.

RPMI-8226 and NCI-H929 cells were transiently transfected with si-CIP2A or si-Scr at different concentrations (25, 50, and 100 nM) and analyzed by qRT-PCR 24 h later.* CIP2A* mRNA expression level in RPMI-8226 cells decreased by 52%, 72%, and 84% after si-CIP2A transfection at 25, 50, and 100 nM, respectively (Figure 1(a)).* CIP2A* mRNA expression level in NCI-H929 cells decreased by 53%, 75%, and 80% after si-CIP2A transfection at 25, 50, and 100 nM, respectively (Figure 1(b)). CIP2A protein expression was examined in cells 48 h after siRNA transfection. The protein level was almost completely suppressed by 100 *µ*M si-CIP2A treatment in both RPMI-8226 and NCI-H929 cells (Figure 1(c)); this concentration was therefore used in subsequent experiments.

### 3.2. CIP2A Silencing Inhibits Multiple Myeloma Cell Proliferation

**Figure 2:**
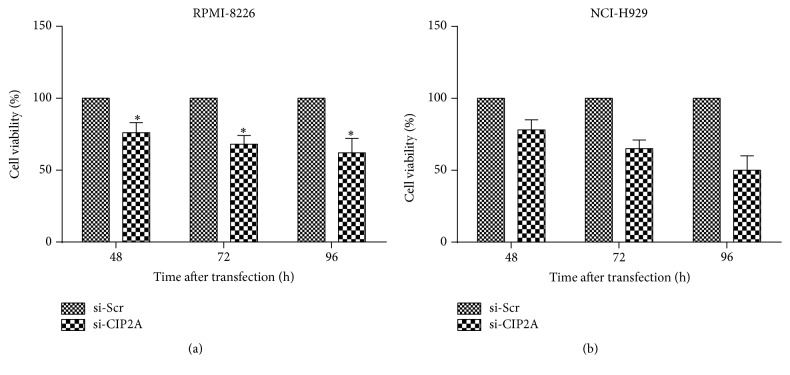
Effect of* CIP2A* knockdown on RPMI-8226 (a) and NCI-H929 (b) cell proliferation. Viable cells were counted 48, 72, and 96 h after transfection. Data are expressed as mean ± SD. ^*∗*^ *P* < 0.05.

The role of CIP2A in human multiple myeloma was investigated by knocking down* CIP2A* expression in RPMI-8226 and NCI-H929 cells and evaluating the effect on cell proliferation. Loss of* CIP2A* expression in RPMI-8226 cells inhibited cell viability by 24%, 32%, and 38% at 48, 72, and 96 h, respectively, after transfection (Figure 2(a)). Loss of* CIP2A* expression in NCI-H929 cells inhibited cell viability by 22%, 35%, and 50% at 48, 72, and 96 h, respectively, after transfection (Figure 2(b)).

### 3.3. CIP2A Inhibition Induces Multiple Myeloma Cell Apoptosis

**Figure 3:**
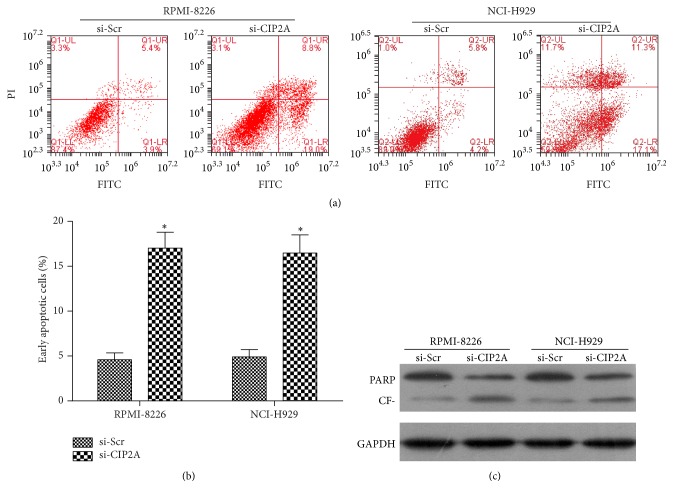
Effect of* CIP2A* knockdown on multiple myeloma cell apoptosis. (a) Representative image of a flow cytometry scatterplot of apoptotic RPMI-8226 and NCI-H929 cells labeled with fluorescein isothiocyanate (FITC) and propidium iodide [13] and sorted 72 h after transfection with si-CIP2A or si-Scr. (b) Quantitative analysis of early apoptotic cells after transfection with si-CIP2A or si-Scr. Data are expressed as mean ± SD. ^*∗*^ *P* < 0.05. (c) The cleavage of PARP detected by western blot. CF: cleaved fragment.

The effect of CIP2A inhibition on apoptosis was examined by flow cytometry. The apoptotic fraction was higher upon transfection of si-CIP2A as compared to si-Scr both in RPMI-8226 and in NCI-H929 cells (Figure 3(a)); specifically, the percentage of early apoptotic cells was higher in cells depletion in* CIP2A* than in control cells both in RPMI-8226 and in NCI-H929 cells (Figure 3(b)). To further evidence whether si-CIP2A induced apoptosis, we detected the cleavage of PARP. As shown in Figure 3(c), the cleavage of PARP was more obvious in cells depletion in* CIP2A* than in control cells both in RPMI-8226 and in NCI-H929 cells.

### 3.4. CIP2A Knockdown Inactivates PI3K/AKT/mTOR Signaling

**Figure 4:**
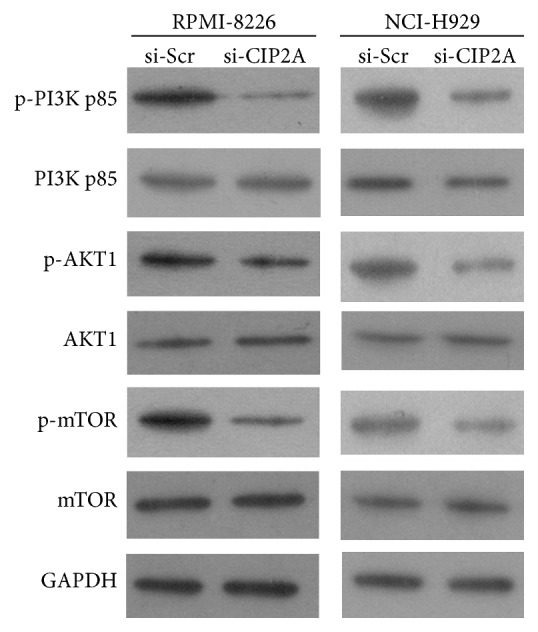
Effect of* CIP2A* knockdown on the expression level of PI3K/AKT/mTOR signaling components. The protein expression levels of nonphosphorylated and phosphorylated PI3K p85, AKT1, and mTOR in RPMI-8226 and NCI-H929 cells transfected with si-CIP2A or si-Scr were evaluated by western blotting, with glyceraldehyde-3-phosphate dehydrogenase (GAPDH) used as a loading control.

To investigate the mechanism underlying the effect of CIP2A on RPMI-8226 and NCI-H929 cell proliferation and apoptosis, we evaluated the expression of key proteins in the PI3K/AKT/mTOR signaling pathway 72 h after si-CIP2A or si-Scr transfection by western blotting.* CIP2A* knockdown decreased the phosphorylation level of PI3K p85, AKT1, and mTOR without altering total protein levels both in RPMI-8226 and in NCI-H929 cells (Figure 4), suggesting that pathway activation but not the expression of individual components was affected by loss of CIP2A.

### 3.5. IGF-1 Treatment Decreases the Effects of CIP2A Knockdown in Multiple Myeloma Cells

**Figure 5:**
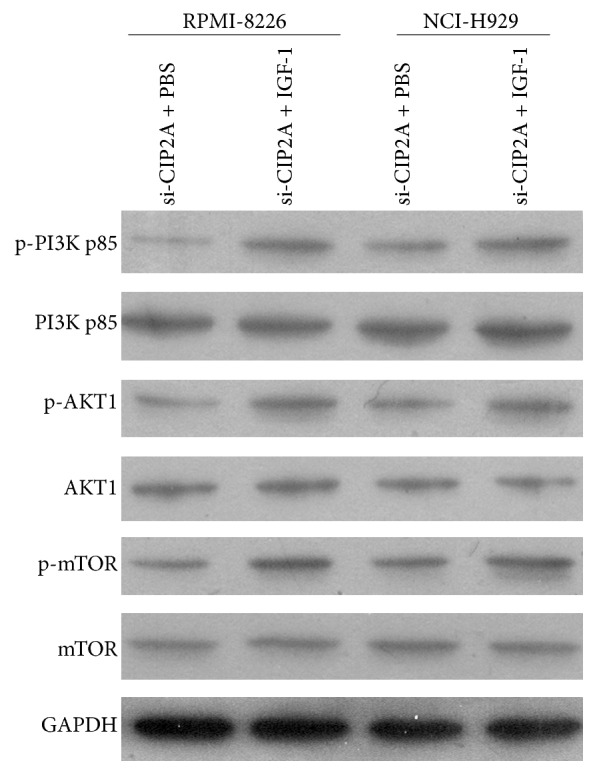
Effect of IGF-1 treatment on the expression level of PI3K/AKT/mTOR signaling components in RPMI-8226 and NCI-H929 cells that are knocked down by si-CIP2A. The protein expression levels of nonphosphorylated and phosphorylated PI3K p85, AKT1, and mTOR in RPMI-8226 and NCI-H929 cells that are knocked down by si-CIP2A treated with PBS or IGF-1 were evaluated by western blotting, with glyceraldehyde-3-phosphate dehydrogenase (GAPDH) used as a loading control.

**Figure 6:**
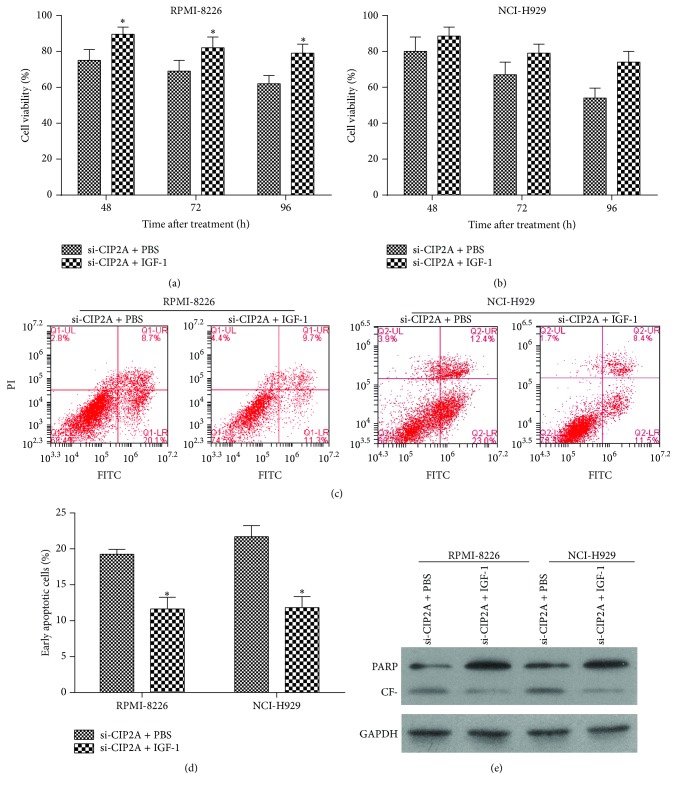
IGF-1 treatment reverses the effects of CIP2A knockdown in RPMI-8226 and NCI-H929 cells. ((a) and (b)) RPMI-8226 (a) and NCI-H929 (b) cells transfected with si-CIP2A were treated with PBS (control) or the PI3K activator IGF-1, and the effect on cell viability was determined by the MTS assay. The results were normalized to si-Scr transfected cells. ((c) and (d)) Effect of IGF-1 treatment on RPMI-8226 and NCI-H929 cell apoptosis. Flow cytometry scatterplot of apoptotic RPMI-8226 and NCI-H929 cells labeled with fluorescein isothiocyanate (FITC) and propidium iodide and sorted 72 h after transfection with si-CIP2A followed by treatment with PBS or IGF-1 (c) and quantitative analysis of early apoptotic cells after transfection with si-CIP2A followed by treatment with PBS or IGF-1 (d). (e) The cleavage of PARP in each group detected by western blot. CF: cleaved fragment. Data are expressed as mean ± SD of three independent experiments. ^*∗*^ *P* < 0.05.

To clarify the role of PI3K/AKT/mTOR signaling in the regulation of multiple myeloma cell proliferation and apoptosis by CIP2A, we evaluated the effect of IGF-1 which could activate PI3K [14] on RPMI-8226 and NCI-H929 cells. Firstly, we tested the expression level of PI3K/AKT/mTOR signaling components upon IGF-1 treatment in RPMI-8226 and NCI-H929 cells that are knocked down by si-CIP2A. As shown in Figure 5, the decreased phosphorylation of PI3K p85, AKT1, and mTOR caused by si-CIP2A was increased by IGF-1 treatment. Then we evaluated the effect of IGF-1 on cell proliferation and apoptosis in RPMI-8226 and NCI-H929 cells that are knocked down by si-CIP2A. The results showed that treatment with IGF-1 (100 ng/mL) reversed the decrease in cell viability caused by* CIP2A* knockdown (Figures 6(a) and 6(b)) and reduced the rate of apoptosis relative to untreated si-CIP2A-transfected cells (Figures 6(c) and 6(d)). In addition, treatment with IGF-1 reversed the increase in the cleavage of PARP caused by* CIP2A* knockdown (Figure 6(e)). Taken together, the results indicate that CIP2A knockdown modulates multiple myeloma cell proliferation and apoptosis via inhibition of PI3K/AKT/mTOR signaling.

## 4. Discussion

CIP2A is a human oncoprotein that was initially identified in patients with gastric and liver cancers, which has unknown function due to the lack of the homology to any known proteins [12]. CIP2A is overexpressed in several types of cancer cell [5, 10, 15–18], and its utility as a prognostic marker has been established in various solid and hematological cancers, including esophageal squamous cell and non-small cell lung carcinoma; breast, gastric, bladder, ovarian, tongue, hepatocellular, and colon cancers; and chronic myelogenous leukemia [5, 10, 15–21]. The present study investigated the role of CIP2A in human multiple myeloma using the RPMI-8226 and NCI-H929 cell lines. We found that* CIP2A* knockdown inhibited proliferation and induced apoptosis, suggesting that CIP2A acts as an oncoprotein in RPMI-8226 and NCI-H929 cells. This is in agreement with results of previous studies; for instance, in gastric cancer, CIP2A was detected in tumor tissues but not in normal gastric mucosa, and depleting* CIP2A* expression suppressed the growth of tumor cell lines [11]. In addition, CIP2A is overexpressed in esophageal squamous cell carcinoma relative to normal tissues, and* CIP2A* knockdown was found to inhibit tumor cell growth [19]. However, our results on the effect of CIP2A on cell apoptosis are inconsistent with results of previous studies; for instance, in cervical cancer, breast cancer, and hepatocellular carcinoma, CIP2A knockdown does not induce significant apoptosis [9, 22, 23]. It is difficult to explain the discrepancy based on the present study. However, all these results showed that CIP2A function is not conserved across different cancer cell types.

Based on the experimental evidence that CIP2A has clinical relevance in the progression of the disease it has been regarded that CIP2A inhibitors have potential for use in the treatment in cancers [24]. So far CIP2A has been targeted in a limited number of cancers, such as hepatocellular carcinoma [25], as well as oral cancer [26]. The expression of* CIP2A* also could be inhibited by natural compounds and fusogenic-oligoarginine peptide-mediated delivery of siRNAs for gene silencing and erlotinib derivatives [24]. And high expression of CIP2A has also been proposed as a useful biomarker that predicts therapeutic response to chemotherapeutics such as bortezomib, erlotinib, afatinib, checkpoint kinase 1 inhibitors, and prosenescence based therapies [13, 27–29]. Based on these previous results, we predicted that CIP2A might serve as a potential target for therapeutic agents developed for the treatment of multiple myeloma.

PI3K/AKT/mTOR signaling plays a critical role in the malignant transformation of human tumors and their subsequent growth, proliferation, and metastasis [30]. In the present study, we found that CIP2A knockdown led to decreased phosphorylation of PI3K p85 at Tyr458, AKT1 at Ser473, and mTOR at Ser2448, and treatment with the IGF-1 which could activate PI3K [14] decreased the effect of CIP2A knockdown on the expression level of PI3K/AKT/mTOR signaling components. CIP2A overexpression can induce an increase in AKT phosphorylation in head and neck squamous cell carcinoma [22] and in liver cancer cells on both Thr308 and Ser473 of AKT; moreover, CIP2A inhibition induced the dephosphorylation of Ser473 in liver cancer and triple-negative breast cancer and lung cancer cells [23, 31, 32]. In addition, the inhibition of PP2A which could be inhibited by CIP2A activates PI3K/AKT signaling through regulating the phosphorylation of AKT at Ser473 in SV40ST-induced human cancer cell transformation [8]. Based on these results, we predicted that Ser473 of AKT is a direct substrate of PP2A in human multiple myeloma using the RPMI-8226 and NCI-H929 cell lines. As an inhibitor of PP2A which is a serine/threonine phosphatase, there is no evidence showing CIP2A can regulate the phosphorylation of PI3K p85 at Tyr458 or PP2A can directly dephosphorylate tyrosine phosphorylation sites of PI3K p85. Therefore, p-PI3K p85 cannot be a direct substrate of PP2A, although phosphorylation of PI3K p85 at Tyr45 could be decreased by CIP2A knockdown. Previous studies indicated the phosphorylation of mTOR at Ser2448 was also not a direct substrate of PP2A [33, 34]. These results indicated that CIP2A could regulate PI3K/AKT/mTOR signaling pathway, but the regulatory mechanisms between CIP2A and PI3K/AKT/mTOR signaling pathway seemed to be complex.

**Figure 7:**
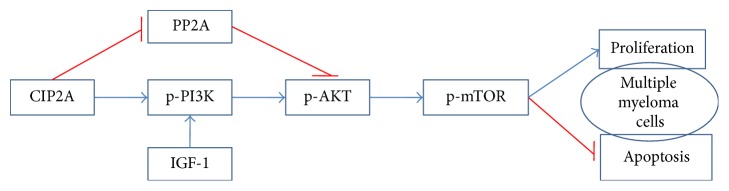
The scheme illustrating the mechanism by which knockdown CIP2A induces apoptosis and antiproliferation including the counteraction of IGF-1 and PP2A in myeloma cells. Blue arrows indicate activation. Red T-bars indicate suppression.

In conclusion, the results of our study indicate that CIP2A acts as an oncoprotein in human multiple myeloma. Knocking down* CIP2A* expression inhibited cell proliferation and induced early apoptosis via inactivation of PI3K/AKT/mTOR signaling, as shown in Figure 7. Therefore, CIP2A can serve as a potential target for therapeutic agents developed for the treatment of multiple myeloma.
